# Cellular Automata Modeling of Silica Aerogel Condensation Kinetics

**DOI:** 10.3390/gels7020050

**Published:** 2021-04-21

**Authors:** Nina H. Borzęcka, Bartosz Nowak, Rafał Pakuła, Robert Przewodzki, Jakub M. Gac

**Affiliations:** Faculty of Chemical and Process Engineering, Warsaw University of Technology, 00-645 Warsaw, Poland; bartosz.nowak.dokt@pw.edu.pl (B.N.); rafal.pakula.stud@pw.edu.pl (R.P.); robert.przewodzki.stud@pw.edu.pl (R.P.); Jakub.Gac@pw.edu.pl (J.M.G.)

**Keywords:** silica aerogels, condensation kinetics, reaction-limited aggregation, nucleation and growth, cellular automata

## Abstract

The formation of silica aerogels and the kinetics of condensation were investigated numerically. The influence of the reaction-limited to the diffusion-limited aggregation (RLA to DLA) transition on the reaction kinetics curves and the evolution of the aggregate size distribution during condensation were examined. The 2D cellular automaton was developed and applied to reflect the process of secondary particle aggregation. Several tendencies were observed due to the adjustment of the model parameters: the probability of condensation reaction and the particles’ concentration. The final wet-gel structures’ visualizations proves that the structure becomes more dense and compact due to entering the RLA regime. The simulation time (associated with the gelation time) decreased along with the increase in both model parameters. The lower the collision probability, the slower reaction becomes, and particles are more likely to penetrate the structure deeper until they finally join the aggregate. The developed model reflects the condensation process’s nature and its mechanisms properly and indicates a significant potential for further aerogel synthesis investigations and for the prediction of wet-gel properties according to condensation parameters.

## 1. Introduction

Silica aerogels have gained increasing attention since they were first synthesized in 1931 [1]. Utilizing alkoxysilanes as precursors, instead of silica oxides, provided control over the final material structure, which is the source of the aerogel’s unique properties. Nonetheless, the formation of aerogel depends on plenty of parameters: temperature, silane/solvent/water molar ratio, catalyst type and its concentration [2,3,4,5,6], to name just a few. The sol-gel procedure conditions lead to changes in the final product features due to differences in the kinetics parameters. The influence of a specific set of parameters remains unclear, and often structures are obtained through trial and error by each group of authors separately. The sol-gel method’s complexity, together with numerous process variables, stems the need for further investigation to better understand aerogel formation’s nature.

In parallel with experimental research, a theoretical and numerical study is being developed, allowing prediction of aerogels’ structure, mechanical and thermal properties and the dynamics of changes in this structure. To date, most of the attention has been directed to the numerical modeling of the aerogels’ structure (the exhaustive review can be found in [7]). Furthermore, much interest was paid to the mechanical properties of aerogels [8]. Among the used numerical methods, it is worth paying attention to molecular dynamics simulations [9,10], which were used to model the aerogels’ mechanical properties. The links between aerogel structures and their mechanical properties have also been analyzed utilizing hard-sphere aggregation models by Ma et al. [11,12]. Other methods used here are coarse-grained models [13,14,15] and multiscale models [7]. The thermal properties of aerogels have been modeled through, e.g., atomistic simulations [16]. The silica aerogels are mainly obtained by the sol–gel process that assembles small and nearly uniform particles into clusters or networks. This mechanism caused cellular automata (CA) [17] or other particle-based algorithms to model aerogel structure and properties. The latter includes, e.g., diffusion-limited aggregation (DLA), reaction-limited aggregation (RLA), diffusion-limited cluster–cluster aggregation (DLCA), reaction-limited cluster–cluster aggregation (RLCA) and a combination of the above. The highest popularity has been reached by the DLCA model [18,19]. DLCA modeling is used to describe, e.g., the structure of an aerogel network [20] and its mechanical deformation [21].

However, despite significant progress in aerogel modeling in recent years, there is still a lack of work describing the kinetics of an aerogel formation. One of a few investigations in this field is our last paper, where we have elaborated a simple kinetics model of the gelation process [22]. The results obtained by employing this model appeared to be in good agreement with experimental results. However, this model gave no insight into an aerogel structure. Other works coped with the kinetics of simple macromolecular structures formation, such as dimers, chains or rings [23,24]. The modelling results are in good agreement with the experimental results of NMR and UV Raman spectroscopy, but they do not allow one to find the complete dependence of gel mass on time.

In the current paper, we propose the two-dimensional particle-based RLA/RLCA and DLA/DLCA modeling of gelation dynamics. We also identify the relations between parameters of the model and parameters of the actual gelation process, and, thus, show the influence of these parameters on the dynamics of gelation kinetics, i.e., the shape of the kinetics curve and the time of gelation. Presented results also give some insight into the morphology and structure of the final product; however, the quantitative information about aerogel structure will provide a 3D model. The future model iteration has a promising potential for further evolution and alterations, taking into account data from experimental techniques, such as SAXS [25] or DLS.

## 2. Results and Discussion

**Figure 1:**
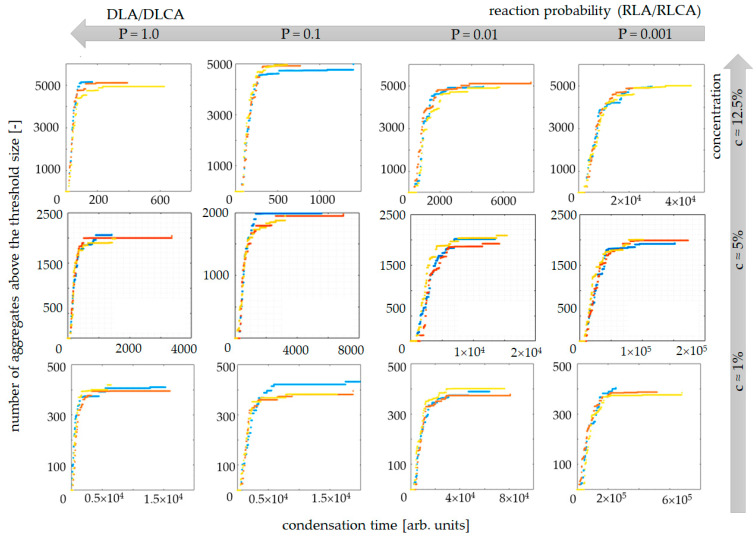
Condensation kinetics curves obtained by cellular automata modeling for different values of reaction probability (*P*) and concentrations of secondary particles (c).

Figure 1 presents an examination of the influence of the DLA/DLCA to RLA/RLCA transition on the numerically designated condensation reaction kinetics curves. The model parameters were (i) concentration of aerogel secondary particles, c, and (ii) probability of condensation reaction, P. Simulations were conducted for three chosen concentration values: c ≈ 1%, 5% and 12.5% and four probability values: *P* = 0.001, 0.01, 0.1 (reaction-limited (cluster) aggregation, RLA/RLCA) and 1 (diffusion-limited (cluster) aggregation, DLA/DLCA). Simulations were conducted three times for every case to verify the results’ repeatability (blue, red and yellow curves, as shown in Figure 1).

The time of a simulation (associated with the moment when 100% of secondary particles are connected within one aggregate, i.e., the structure is completely cross-linked) decreases as the system approaches the DLA/DLCA regime due to the higher probability of reaction between two adjacent particles/clusters. The same tendency occurs with an increase in secondary particle concentrations. A higher amount of particles within the computing domain is also responsible for a higher probability of collision between particles/clusters for the same reaction probability value.

**Figure 2:**
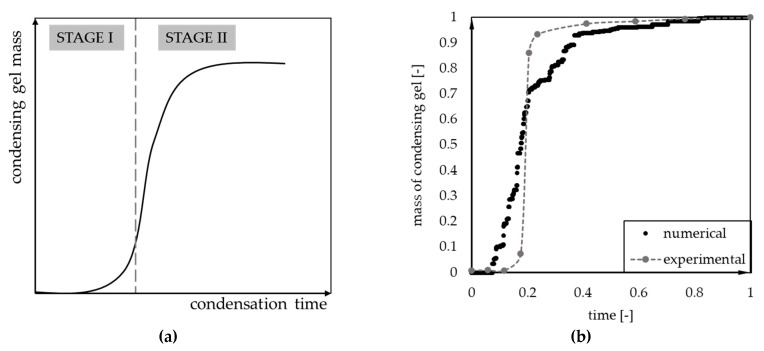
(**a**) The assumed division of condensation kinetics curves into two stages and (**b**) qualitative comparison of experimental [22] and numerical condensation kinetics curve.

The final number of aggregates above the threshold size (0.5% of the total number of cells) is associated with a mass of condensing aerogel. The two alternative methodologies for the experimental designation of condensation kinetics was thoroughly described in our previous paper [22]—via the filtration of gelling solution and determination of the resulting product mass or via the UV–Vis spectrophotometry. Both methods provided the sigmoidal shape of kinetics curves for both samples with or without macroscopic phase separation and “particle aggregates” type of microstructure [26]. We identified two stages and the prevailing mechanisms of aerogel condensation kinetics (Figure 2a). During the first stage of condensation kinetics, the dominant mechanism is the reaction between free molecules of the precursor or aggregates containing few molecules. In the second stage, a surface reaction appears between single precursor molecules and already formed aerogel spheres. The merging of bigger (already “detectable”) clusters was not considered, as it do not affect the mass of the condensing product.

Comparing the previously obtained results (experimental and numerical) with the ones obtained by cellular automata modeling, one can notice that the first condensation stage is not especially evident. It may result from too small a computing domain (more information in Section 4: Materials and Methods). Otherwise, to capture the initial stage of condensation kinetics a different method must be proposed, reflecting primary particles’ formation due to the polycondensation reaction between precursor molecules (e.g., the coarse-grained molecular dynamic). The superposition of two numerical methods shows the potential to thoroughly reflect the reaction kinetics and provide an insight (visualization) into the process.

Figure 2b presents preliminary comparison of the numerically obtained kinetics curve (model parameters: *P* = 0.01, c = 12.5%) and experimental measurements. The chemical composition of the sample was 1:2:0.6:0.6 (vol. MTMS: MeOH: oxalic acid 0.01 M: ammonia 1 M) [22]. The results were normalized to the maximum value of condensing aerogel mass and total measurement time. The comparison indicates a similarity between the shape of experimental and numerical curves.

**Figure 3:**
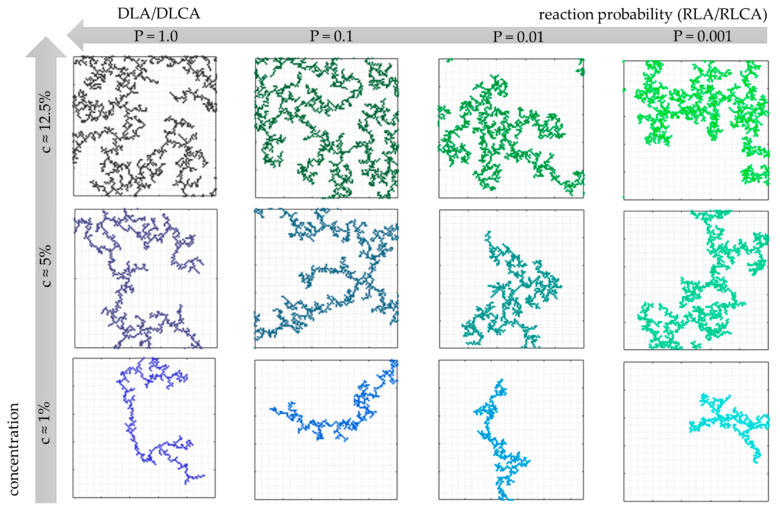
Final wet–gel structure visualizations obtained by cellular automata modeling for different values of reaction probability and concentrations of secondary particles.

Figure 3 presents the visualizations of the final structures obtained by the cellular automata modeling for the chosen values of reaction probability and concentration. For each concentration value (c ≈ 1%, 5% and 12.5%), one can notice that the structure becomes more dense and compact along with the decrease in the condensation reaction probability value. This is a consequence of reaction-limited aggregation—the probability of merging particles/clusters is significantly lower. Thus, the particles are more likely to penetrate the structure deeper until they join the aggregate at some point.

**Figure 4:**
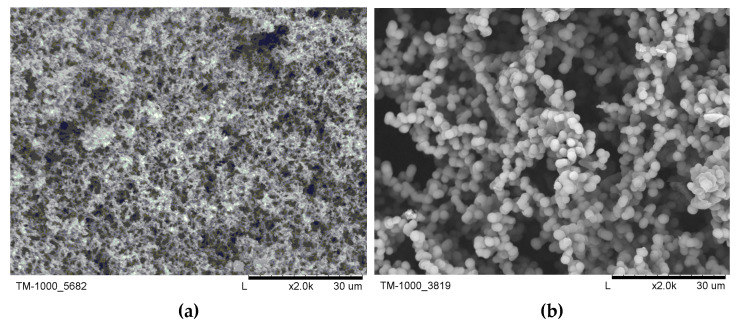
SEM images of exemplary aerogel structures. Chemical compositions (vol. MTMS: MeOH: oxalic acid 0.01 M: ammonia 1 M) (**a**) 1:2:0.6:0.6 and (**b**) 1:2:0.8:0.8. Magnification 2000× [22].

Figure 4 shows exemplary SEM (scanning electron microscopy) structures obtained experimentally during our previous research [21]. The particle aggregates type of structure [26] resembles the character of the numerically obtained wet-gel structures (Figure 3).

**Figure 5:**
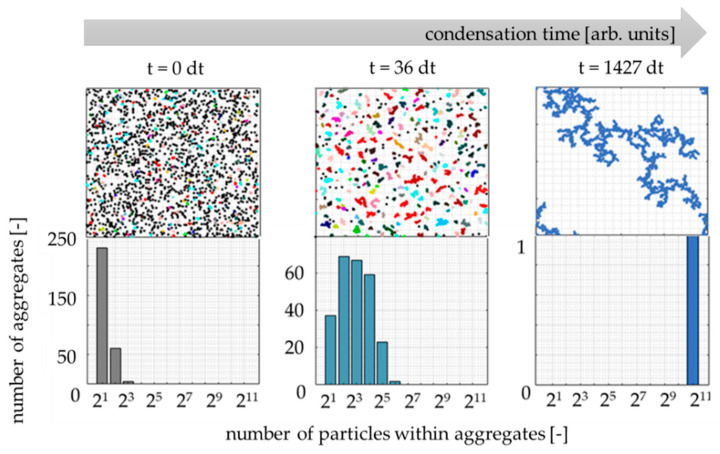
The evolution of the aggregate size distribution during condensation (c = 5%, *P* = 1). Colors of aggregates were randomly generated for easy distinction.

The developed model also provides the aggregates’ size distribution (dependent on the number of particles within the aggregates) during the condensation time. The exemplary results are presented in Figure 5. At the system’s initial state (t = 0), the vast majority of particles are single (not connected). However, some particles already merge at the stage of random generation of their initial location. During the condensation, the total number of aggregates decreases, and the distribution moves towards bigger aggregates until the system is entirely cross-linked (t = 1427 dt).

As the presented studies’ main goal is to develop a model reflecting the condensation kinetics, the gelation time value was compared for both experimental and numerical methods.

**Figure 6:**
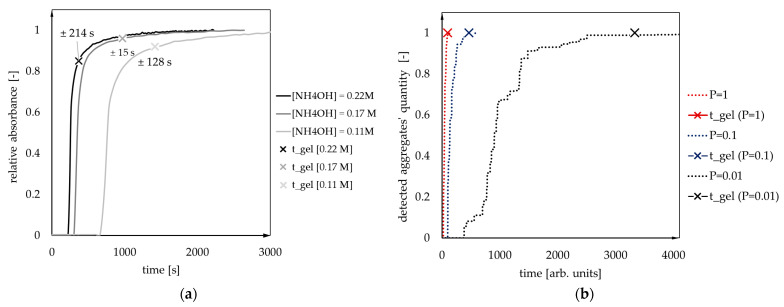
The condensation kinetics curves obtained (**a**) experimentally (UV–Vis spectrophotometry) and (**b**) numerically. The gelation time values (cross points) estimated by (**a**) tilting test tube method and (**b**) assuming the gelation time point occurs for structure cross-linked in 99%.

Figure 6a shows the condensation kinetics obtained by the UV–Vis spectrophotometry for samples with variable ammonia concentration (0.11, 0.17 and 0.22 [mol/dm^3^]) and constant MTMS/MeOH and acid/base molar ratios (more information in Section 4: Materials and Methods). The cross points marked on the kinetics curves stand for the gelation time values, estimated by the tilting test tube method. It was noticed that gelation occurs approximately right after the inflexion of a kinetics curve.

A similar tendency was observed for numerical results (Figure 6b) when the gelation time was assumed at the point when the structure is cross-linked in 99% (which means that 99% of secondary particles are a part of a detectable aggregate).

**Figure 7:**
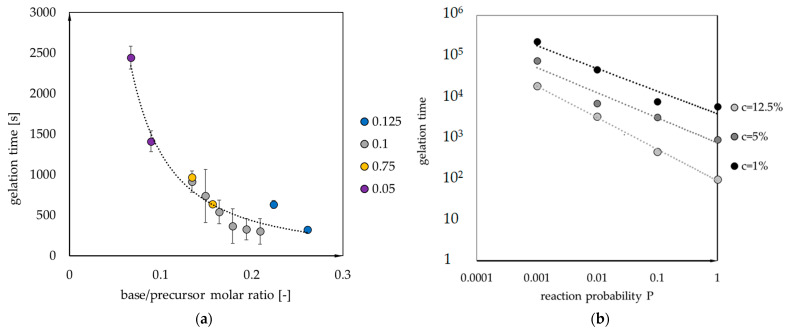
The dependence of (**a**) gelation time on base/precursor molar ratio (for different base/acid ratios, marked with different point colors) and (**b**) logarithm of gelation time on the reaction probability logarithm.

Figure 7 presents the analysis of the reaction probability’s influence—assumed to be dependent on base catalyst concentration. Experimentally designated values of gelation time for samples with a variable base/precursor ratio are presented in Figure 7a. The dependence can be approximated by the power function:

(1) $$t_{gel}=36.2\cdot {\left(n_{NH4OH}/n_{MTMS}\right)}^{-1.5}$$

With coefficient R^2^ = 0.85.

Power function can also approximate the dependence of gelation time on reaction probability (Figure 7b):

(2) $$t_{gel}=A\cdot P^{n}$$

The values of parameters in the above formula are given in Table 1.

By comparing Equations (1) and (2), we may observe that reaction probability *P* as a model parameter is a growing function of molar ammonia to MTMS ratio. More precisely, it reads:

(3) $$P\propto {\left(n_{NH4OH}/n_{MTMS}\right)}^{\alpha }$$

where the exponent α=−1.5n equals between 2 and 3, dependent on the concentration of the secondary particles. By comparing (3) with (8), we may deduce how the ammonia to MTMS molar ratio lowers the activation energy:

(4) $$E_{a}\propto -ln\left(n_{NH4OH}/n_{MTMS}\right)$$

## 3. Conclusions

The developed model, despite its simplicity, reflects the nature of the condensation process properly. The final structures become denser while the system is approaching the RLA/RLCA regime. The shape of the kinetics curve obtained from a numerical model was similar to that obtained from experimental investigations. The time of gelation decreased with the increase in reaction probability value and the increase in secondary particle concentrations, which itself increased the likelihood of a collision.

The gelation time dependence on the reaction probability (numerical results) and the concentration of the base catalyst (experimental) were approximated by power functions. The gelation time happened right after the inflexion of the experimental kinetics curve. This observation stays in good correspondence with numerical results with gelation time assumption as the 99% extent of cross-linking. Furthermore, it was deduced that the ammonia to MTMS molar ratio lowers activation energy, according to the Equation (4).

The model indicates a significant potential for further aerogel synthesis investigations and predictions of wet-gel properties according to condensation parameters. Further iteration requires the transition into the 3D system, while the present 2D model does not describe the complexity of the 3D aerogel structure.

The next step should be correlating the size of an aggregate with its diffusivity (utilizing, e.g., Einstein–Stokes equation), thus the frequency of motion in the CA system. Another factor that should be considered is the condensation temperature, affecting the reaction mixture’s viscosity and particles’ diffusivity.

## 4. Materials and Methods

### 4.1. Cellular Automata Studies

**Figure 8:**
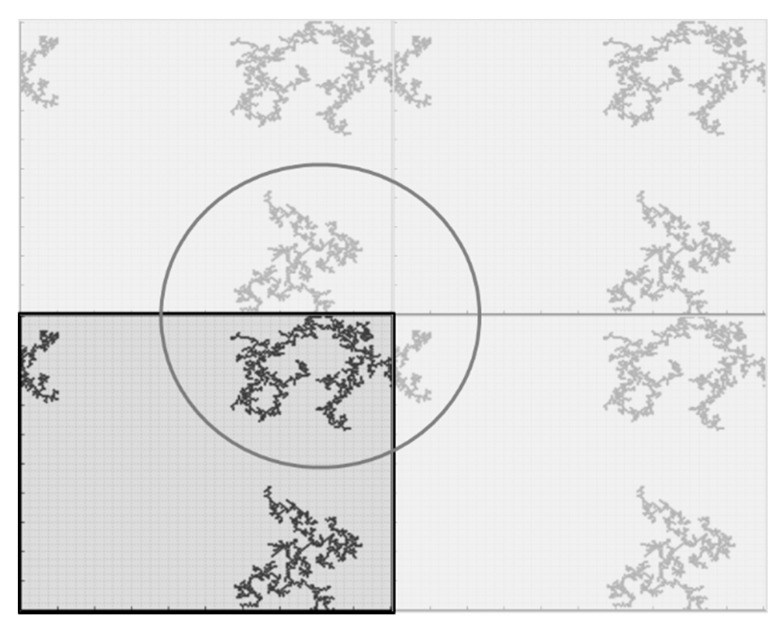
Scheme of periodic boundary conditions.

The two-dimensional computing domain representing the condensing mixture was divided into a regular grid of cells with periodic boundary conditions (Figure 8). Each cell can be described by one of a finite number of states—in this particular case, we only had two possible states: (i) a cell was occupied by a secondary particle or (ii) the cell was empty. According to the set percentage concentration value (assumed to be a ratio of occupied to total number of cells), the number of secondary particles was given as one of the model parameters. An initial state of the system (for t = 0) was pseudo-randomly generated. According to the set reaction probability value, if two particles meet in the adjacent cells (the Moore type of neighborhood was applied), the reaction of condensation can occur. The reaction probability is a significant model parameter deciding whether aggregation is limited by diffusion (*P* = 1, DLA/DLCA) or reaction (*P* < 1, RLA/RLCA). If the reaction between adjacent cells does indeed occur, the two particles start to act as one aggregate. Particles belonging to different aggregates may also join each other if they appear in neighboring cells. The probability of reaction is assumed to be the same as for free particles. The reaction between particles that already belong to aggregates results in the aggregate growth according to the DLCA or RLCA mechanism, depending on *P* value.

The probability *P* is dependent on the kinetics constant of the condensation reaction. Indeed, assuming that two clusters may join in every time step Δ*t* with probability *P,* we conclude that in every time step there appears to be a number of newly formed aggregates *N_new_* given as:

(5) $$N_{new}=P\cdot N_{coll}$$

where $N_{coll}$ is a number of colliding pair of aggregates and is proportional to the square of aggregates (or secondary particles) concentration $N_{coll}\propto c^{2}$. Thus,

(6) $$N_{new}=\alpha \cdot P\cdot c^{2}$$

where α is a proportional coefficient, and the condensation rate is given as:

(7) $$v≅\frac{N_{new}}{∆t}=\alpha \frac{P}{∆t}\cdot c^{2}$$

On the other hand, the kinetics theory of chemical reaction reads as follows:

(8) $$v=k\times c^{2}$$

Thus, we have $k\propto \frac{P}{∆t}$.

Following the above, probability *P* depends on the activation energy, temperature and collision effectiveness. In the same manner as *k*, *P* is the increasing function of collision effectiveness and temperature and a decreasing function of activation energy in the form of

(9) $$P\propto exp\left(-\frac{E_{a}}{k_{B}T}\right)$$

where *k_B_* is a Boltzmann constant.

In our parametric study, *P* was the first parameter of our model.

The second parameter of the model was a relative concentration of occupied cells (which does not change during the simulation) and should be interpreted as a volumetric concentration of precursor particles.

During the simulation, the particles started to diffuse in random directions, with the total probability of the movement equal to one in every time step. The direction of the motion was chosen randomly with uniform probability 0.125, which means that the sedimentation was not considered. Indeed, for objects as small as secondary particles or small aggregates, diffusion was the dominant mechanism of motion. If the particle was a part of an aggregate, the rest of the particles forming that aggregate moved in the same direction as the first one. This means the clusters’ diffusivity was assumed to be not related to their size, which is significant but (at this point of research) an intended simplification. Another simplification arising from these rules of motion is that the aggregates did not change their shape (e.g., as a result of deflection). For relatively small aggregates, this assumption seems to be valid. The computation procedure stops at the point of complete cross-linking, i.e., when all secondary particles were connected, forming one giant aggregate as presented on the video provided in the Supplementary Materials (Video S1). The animation shows an exemplary simulation for DLA mechanism (concentration = 1%, probability of reaction = 100%). The particles represent aerogel secondary particles. The animation shows their motion and aggregation, until the point when the structure is 100% cross-linked (all particles are connected).

**Figure 9:**
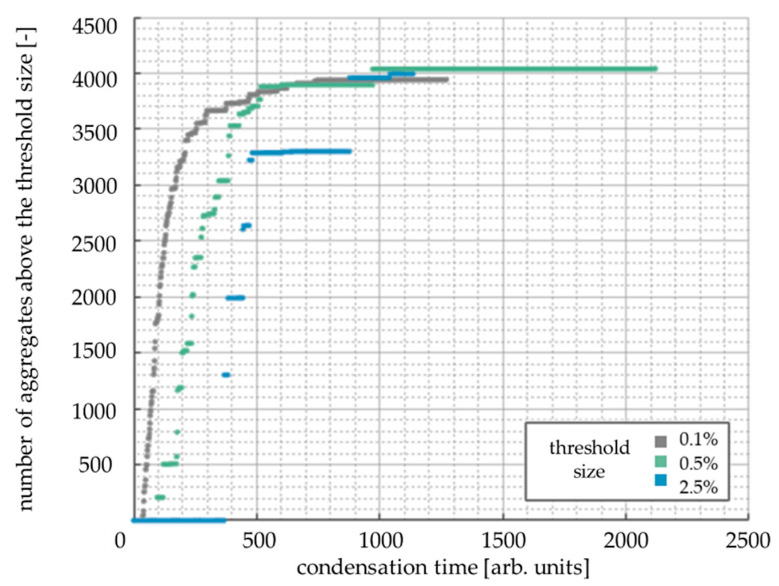
The influence of the threshold aggregate size on the shape of kinetics curves. Secondary particles concentration: 10%, reaction probability *P* = 10%, grid size: 200 × 200 cells.

For the experimental and numerical kinetics curves comparison, it was assumed that an aggregate could be detected when it reached some threshold size. This threshold corresponds to the minimum weight that is visible during the measurements. Three sizes (aggregates consisting of 0.1, 0.5 or 2.5% of the total number of cells) were tested (Figure 9). The curve for 2.5% was not smooth enough to obtain results corresponding to the experimental data. This work shows the results obtained for the value of 0.5%.

**Figure 10:**
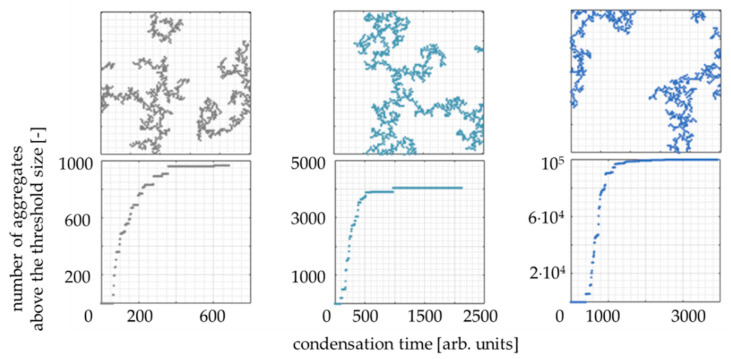
The influence of the threshold aggregate size on the shape of kinetics curves. Secondary particles concentration: 1%, reaction probability *P* = 10%, grid size: 200 × 200 cells.

Three sizes of the computing domain were tested: 100 × 100, 200 × 200, 1000 × 1000 cells. The condensation kinetics curves and final structure visualizations are presented in Figure 10. As far as the final structures are concerned, there is no significant impact of the domain size on the gel morphology. However, the kinetics curves stronger resemble the sigmoidal characters for bigger computing domain sizes. To reflect the kinetics curves’ character, observed with UV–Vis spectroscopy, and to limit the computing power, the 200 × 200 grid was chosen.

For the numerical description of the condensation step of silica aerogels’ synthesis, the C++ programming language was applied, and the MATLAB software was used for the graphs’ preparation. The code was included in the Supplementary Materials (Code S1).

### 4.2. Experimental

The investigated samples were prepared by a two-step (acid-base) sol–gel method. Trimethoxymethylsilane (MTMS) was used as a precursor for the developed synthesis (purchased from Sigma-Aldrich) and methanol (purchased from Stanlab) as a solvent. As acid and base catalysts, the ammonia water (Eurochem BGD) and oxalic acid (Sigma-Aldrich, 0.01 M aqueous solution) were applied.

The syntheses were conducted according to molar ratios provided in Table 2. The oxalic acid solution (0.01 M) was added to start the hydrolysis of MTMS dissolved in methanol. After 1 h, a certain amount of ammonia solution was added. The sample was stirred vigorously for another 30 s, and 2.5 mL of the reaction mixture was transferred into a polystyrene cuvette and placed into the spectrophotometer calibrated on pure methanol. The rest of the sample was used to observe and estimate the time of gelation value by a simple tilting test tube method.

For experimental designation of condensation kinetics (for samples No. 6, 7 and 8), a UV–Vis spectrophotometer (Genesys 10 S UV–Vis spectrophotometer, Thermo Scientific, Waltham, MA, USA) was used at wavelength λ = 633 nm with time intervals (the 20 s for a sample with 0.22 M ammonia solution concentration, 60 s for samples with 0.17 and 0.11 M), until the absorbance remained approximately constant. The absorbance increase in time is proportional to the increase in obtained gel mass during the condensation step of aerogel synthesis [22]. The time of gelation and kinetics for each sample were measured 3 times for each sample and averaged.

## Figures and Tables

**Table 1:** The parameters describing the power dependence of gelation time on the reaction probability.

Secondary Particles Concentration (%)	A Parameter	n Parameter	R^2^
1	3901.8	−0.56	0.94
5	761.8	−0.61	0.95
12.5	90.1	−0.77	1

**Table 2:** The chemical composition and the values of gelation time for the investigated samples.

Sample Nr	Molar Ratio					Conc. NH_4_OH [mol/dm^3^]	Gel. Time [s]	S.dev [s]
	MTMS	MeOH	H_2_O	Oxalic Acid	NH_4_OH			
1	1	7.36	14.87	1.34	0.13	0.19	913.67	129.62
2	1	7.36	14.93	1.34	0.07	0.09	2445.00	141.07
3	1	7.36	16.52	1.49	0.15	0.20	737.67	329.82
4	1	7.36	18.17	1.64	0.16	0.21	540.67	147.78
5	1	7.36	19.78	1.79	0.22	0.28	634.67	49.00
6	1	7.36	19.82	1.79	0.18	0.22	366.00	214.00
7	1	7.36	19.86	1.79	0.13	0.17	966.67	15.00
8	1	7.36	19.91	1.79	0.09	0.11	1411.67	128.00
9	1	7.36	21.47	1.94	0.19	0.23	328.00	129.90
10	1	7.36	23.08	2.09	0.26	0.30	324.00	7.94
11	1	7.36	23.12	2.09	0.21	0.24	301.67	158.66
12	1	7.36	23.17	2.09	0.16	0.18	640.00	17.32

## Supplementary Materials

The following are available online at https://www.mdpi.com/article/10.3390/gels7020050/s1, Video S1: animation DLA, conc = 1%, Code S1: program code.
